# Supplementary material for: Comparative analysis of fecal DNA viromes in Large-billed crows and Northern ravens reveals diverse viral profiles
Source: PeerJ. 2025 Oct 15;13:e20170. doi: 10.7717/peerj.20170 (PMC12535234; doi:10.7717/peerj.20170)
Supplement: Supplemental Information 7 — The number of virus candidate sequences identified by different software: IMG identified 1,200 candidate sequences, Virome Sorter identified 29,795 candidate sequences, VIBRANT identified 20,446 candidate sequences, DeepFinder identified 27,127 candidate sequences. [file peerj-13-20170-s007.pdf]

Comparative analysis of gut DNA viromes in the Large-billed crows and Northern ravens reveals diverse viral profiles

Supplementary Materials

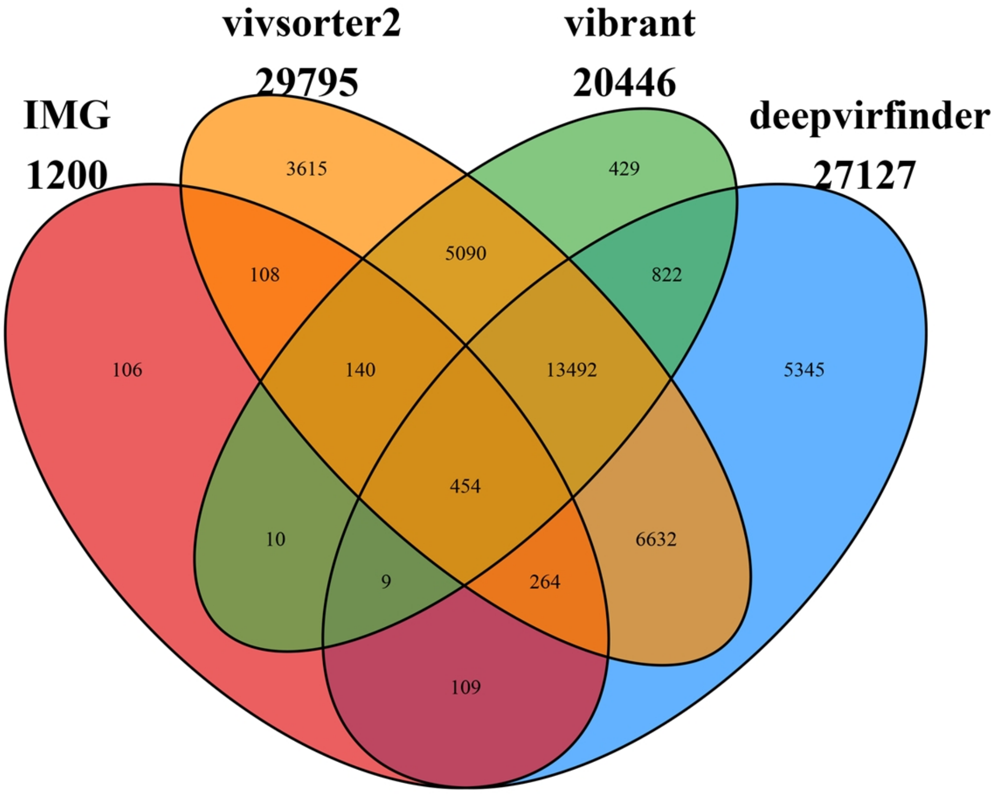

Fig S1: A Venn diagram shows the number of viral candidate sequences identified by IMG, deepVirFinder, VirSorter, and Vibrant
