# Supplementary material for: Comparative analysis of fecal DNA viromes in Large-billed crows and Northern ravens reveals diverse viral profiles
Source: PeerJ. 2025 Oct 15;13:e20170. doi: 10.7717/peerj.20170 (PMC12535234; doi:10.7717/peerj.20170)
Supplement: Supplemental Information 8 — The sequence quality assessed by CheckV, where different colors represent the percentage of sequences at different quality levels, including high quality (3.51%), medium quality (7.17%), low quality (61.63%), and so on. [file peerj-13-20170-s008.pdf]

Comparative analysis of gut DNA viromes in the Large-billed crows and Northern ravens reveals diverse viral profiles

Supplementary Materials

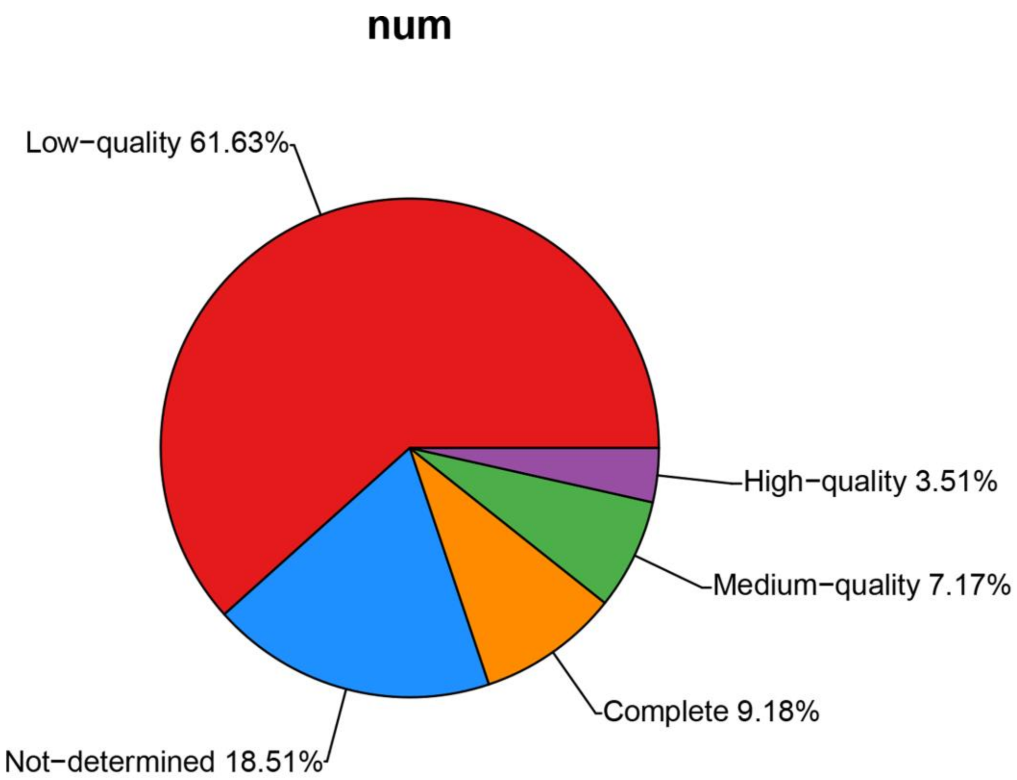

Fig S2: A pie chart shows the quality and completeness of vOTU sequences evaluated by CheckV.
